# Foreign body reaction: towards a macrophage-centered adverse outcome pathway for fibrotic encapsulation

**DOI:** 10.3389/ftox.2026.1735871

**Published:** 2026-02-04

**Authors:** Tom Meseberg, Susanne Kurz, Juliane Spohn

**Affiliations:** Bio- and Nanotechnology Department, Fraunhofer Institute for Ceramic Technologies and Systems (FHG), Dresden, Germany

**Keywords:** adverse outcome pathway (AOP), biomaterials, foreign body reaction, immunotoxicology, ISO 10993, macropage, medical devices

## Abstract

The foreign body reaction (FBR), characterized by chronic inflammation and fibrotic capsule formation around implanted medical devices, remains a major cause in device-related complications. Current preclinical risk assessment relies on *in vivo* testing according to ISO 10993-6:2024, which are limited by species differences, incomplete mechanistic insight, and ethical concerns. Additionally, ISO/TS 10993-20:2006 outlines immunotoxicity knowledge regarding implant-induced effects such as FBR and specifies a collection of *in vitro* assays. The perspective presented here, aims to explore the applicability of the Adverse Outcome Pathway (AOP) framework to FBR in order to integrate evidence and methods into a structured mechanistic context and facilitate the application of *in vitro* tests in preclinical risk assessment of FBR. A targeted literature review was conducted to identify and organize biological mechanisms into a putative AOP, map available new approach methodologies, and highlight critical knowledge gaps and uncertainties. This initial framework may guide early screening for low-FBR materials and support mechanistically anchored, non-animal biocompatibility assessment strategies for medical devices.

## Foreign body reaction to medical devices

The foreign body reaction (FBR), characterized by chronic inflammation and fibrotic capsule formation around implants, remains a major cause of device-related complications. While all materials induce a fibrotic host response after implantation (Anderson et al., 2008; Carnicer-Lombarte et al., 2021), the causes of excessive capsule formation and the impact of material modifications remain debated.

Mechanistically, the FBR begins with protein adhesion to the biomaterial surface, shaping cellular interactions. Monocyte-derived macrophages, with other immune cells, are activated and coordinate acute inflammation, tissue clearance, and early wound healing. When clearance of nondegradable foreign material is frustrated, adherent macrophages fuse into foreign body giant cells (FBGCs), sustaining the inflammatory milieu. Persistent macrophages at the implant site and elevated growth factor signaling promote myofibroblast activation, collagen deposition, and ultimately fibrotic encapsulation (Anderson et al., 2008; Chandorkar et al., 2019).

Across device classes, encapsulation has distinct adverse effects: Greater capsule thickness increases the likelihood and severity of capsular contracture in breast implants, distorting surrounding tissues, impairing implant mechanics, and causing pain (Bachour et al., 2018; Headon et al., 2015; Kim et al., 2022; Larsen et al., 2024). In neural and cochlear implants, “shielding” means that the capsule insulates electrodes from tissue and raises impedance, compromising signal quality and stability (Lotti et al., 2017; Hu et al., 2024; Foggia et al., 2019; Simoni et al., 2020). For implanted biosensors (e.g., glucose monitors), capsules impair diffusion, limiting analyte quantification and long-term reliability (McClatchey et al., 2019; Novak and Reichert, 2015). These results show that implants classified as biocompatible can be affected by FBR.

This raises the question: What does biocompatibility mean immunologically? Widely used medical-device biomaterials today are immunologically bioinert or biotolerable (Rostam et al., 2015; Ratner, 2016). However, because immunogenic response and subsequent tissue integration depend on clinical context, certain applications require a shift from passively bioinert to immunomodulating biomaterials that actively guide physiological wound healing and remodeling (Williams, 2008). Thus, evaluating fibrotic capsule formation as an adverse FBR outcome is important for functionality and safety testing of certain implantable devices in biocompatibility assessment.

## Prediction of foreign body reaction in biological risk assessment

*In vivo* testing according to ISO (2016) is the gold standard for preclinical risk assessment of medical devices and predicting material-induced FBR. Fibrotic capsule formation and post-implantation inflammation are assessed by immune cell counts (macrophages, multinucleated cells) and capsule thickness in histology. While clinically relevant, these models are limited by species differences, mechanistic understanding, and ethical considerations.

ISO (2006) outlines immunotoxicity knowledge on implant-induced effects such as FBR. It identifies implantation-site histopathology per ISO (2016) as the most direct method to assess FBR and chronic inflammation. According to ISO (2006), *in vitro* assays lack intact immune-system complexity yet remain valuable for mechanistic studies. Hence, the tests and indicators listed (e.g., phagocytosis, antigen presentation, cytokine release, MHC phenotyping) remain an unstructured collection with limited predictivity.

Integrating these principles and methods into a structured mechanistic framework enables broader use of *in vitro* data for FBR risk assessment. The Adverse Outcome Pathway (AOP) concept, developed in chemical toxicology, is a systematic, data-driven method linking key events to an adverse outcome. For skin sensitization, ISO (2021) Annex C describes a defined approach combining assays along an AOP with four key events. Comprehensive validation studies on applicability to medical devices are promising but ongoing (Svobodová et al., 2021).

**FIGURE 1:**
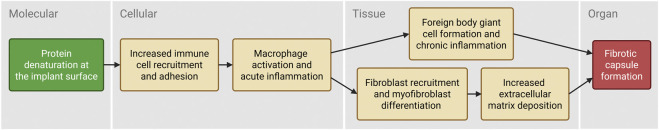
Novel putative adverse outcome pathway (AOP) for mechanism-driven testing of FBR outcome. Created with Biorender.

This perspective explores AOP applicability to FBR to support mechanistic, human-relevant testing strategies. Applying the AOP framework to material-induced reactions is novel and underexplored, offering potential to advance material and nanomaterial risk assessment. A comprehensive search of FBR literature was conducted using PubMed and Scopus. Information was analyzed to identify and organize biological mechanisms into key events. Evidence correlating each key event with fibrotic capsule formation was extracted from selected *in vivo* studies. New approach methodologies relevant to each key event were identified when available. This putative AOP (Figure 1) organizes FBR data, highlighting well-characterized mechanisms and knowledge gaps.

## Molecular initiating event: protein denaturation at the implant surface

After implantation and tissue injury, plasma and extracellular matrix (ECM) proteins adsorb to the implant surface. Biomaterial properties (wettability, topography, hydrophilicity, surface charge) determine adsorption and denaturation extent (Lehká et al., 2023). Denatured ECM proteins expose adhesive motifs, such as RGD, that promote cell adhesion (Swartzlander et al., 2015). Damage-associated molecular patterns (DAMPs) and ECM proteins (e.g., fibrinogen, periostin) regulate immune-cell responses (Franz et al., 2011; Blackman et al., 2024).

A prominent example is denatured fibrinogen, which exposes two neo-epitopes and activates phagocyte pro-inflammatory state via Mac-1 (Hu et al., 2001). Fibrinogen-deficient C57BL/6J mice showed reduced fibrous capsule formation after intraperitoneal or subcutaneous polytetrafluoroethylene implantation (Balabiyev et al., 2021). Similarly, periostin-knockout mice with subcutaneous silicone implants have decreased capsule thickness (Bae et al., 2018). Activation of Toll-like receptors by DAMPs has comparable downstream effects in FBR, as TLR2/TLR4-knockout mice develop thinner capsules after polyethylene glycol hydrogel implantation (Thompson et al., 2025).

Opsonin-mediated recognition (IgG, C3b) likely induces innate immune responses (Anderson et al., 1996), although hypogammaglobulinemic mice showed unaffected neutrophil and monocyte recruitment (Kyriakides et al., 2022).

Lehka et al. introduced an *in vitro* model to evaluate protein adsorption and cell adhesion on biomaterials (Lehká et al., 2023). They reported that adsorbed protein quantity did not determine long-term cell adhesion leading to FBR, whereas dissolution rate was critical. Although data gaps remain and links between protein adsorption and FBR outcomes are debated, growing evidence indicates protein denaturation is crucial. Conversely, plasma treatment enabling covalent attachment of native host proteins reduced capsule formation after subcutaneous implantation of polyurethane in mice (Kondyurina and Kondyurin, 2023).

## Key event 1: immune cell recruitment and adhesion

The interaction of host immune cells with biomaterials, particularly macrophages, is the first key event. Macrophage adhesion and spreading at the material–tissue interface initiate activation. Their activity with effector cells such as fibroblasts is required for capsular fibrosis (Robotti et al., 2018; Brown et al., 2012).

Macrophage depletion with clodronate liposomes reduced capsule formation after subcutaneous polycaprolactone implantation in C57BL/6 mice (Dondossola et al., 2016) and decreased capsule thickness in another study (Mooney et al., 2010). In Rag2/γ KO mice, macrophage depletion eliminated implant-induced fibrosis after alginate implantation (Doloff et al., 2017). Other immune cells appear less critical for FBR in knockout models. T cells (Rodriguez et al., 2009; Doloff et al., 2017), natural killer cells (Yang et al., 2014), and mast cells (Yang et al., 2014; Avula et al., 2014) showed limited or no essential roles. A data gap remains for B cells, as knockout (IghMnull) mice showed only partial reduction in fibrosis (Doloff et al., 2017).

Evidence indicates that integrin-mediated macrophage adhesion affects recruitment and activation. In a rat cage implant system, zwitterionic and anionic polyurethane chemistries reduced nonspecific adhesion, decreased inflammatory events, and promoted immune-cell apoptosis (Khandwekar and Rho, 2012). CD11b knockout and inhibition of RGD-binding integrins reduced capsule thickness around subcutaneous polyethylene terephthalate implants by 30%–45% (Eslami-Kaliji et al., 2023). In MyD88-deficient mice, reduced inflammatory-cell recruitment, mostly macrophages, correlated with a substantial decrease in capsule formation (Amer et al., 2019).

Evidence suggests a “sweet spot” for adhesion to the biomaterial surface. Sustained viable attachment maintains chronic responsiveness to the foreign body (Jannasch et al., 2017a). Too much adhesion may overactivate myofibroblasts, whereas too little adhesion may elicit strong inflammation; both outcomes are detrimental to implant integration (Noscovikova et al., 2021a).

*In vitro* approaches include cell adherence assays on defined surface chemistries with May–Grünwald/Giemsa staining (Chang et al., 2008; Jones et al., 2007; Al-Khoury et al., 2019). Additional assays quantify adhesion across materials using CD54 and beta-actin staining to assess activation-associated adhesion and cytoskeletal organization (Jannasch et al., 2017b).

## Key event 2: macrophage activation and acute inflammation

Macrophage activation is required for FBR-associated fibrosis. However, the host macrophage response is essential for constructive tissue remodeling following implantation (Brown and Badylak, 2013). In physiological wound healing, macrophages shift from M1 to M2a-like during days four to seven post-injury (Witherel et al., 2019). Eliminating M1 signaling or prematurely promoting M2a is linked to excessive ECM deposition and pathological fibrosis, underscoring that polarization timing and dynamics are critical (Saleh et al., 2022). An optimal implant elicits a mild initial M1 response followed by early restricted M2 dominance (Klopfleisch, 2016).

After abdominal implantation of mesh materials in Sprague–Dawley rats, the day-14 M2:M1 ratio correlated with day-35 histological FBR scores (Brown et al., 2012; Wolf et al., 2014). In BALB/c mice, polymers that modulate macrophage polarization showed a balanced M1:M2 ratio which was associated with reduced capsule thickness compared with M1 or M2 predominance (Rostam et al., 2020). Modified polyurethane-coated implants reduced capsule thickness in Sprague–Dawley rats, correlated with increased M2:M1 ratio (CD86 vs. CD163) at 4 weeks (Huang et al., 2018). The macrophage M1:M2 ratio (CCR7+ vs. Arg-1+) correlated with capsule thickness after subcutaneous implantation of functionalized hernia mesh in Sprague–Dawley rats (Yang et al., 2025). Consistent with fibrous encapsulation as a wound-healing response that fails to resolve, the M2:M1 ratio is indicative in other chronic conditions, including spinal cord injury, chronic wounds, and inflammatory renal disease (Yu et al., 2016).

Data gaps remain. Macrophages at implant interfaces can display features of both M1 and M2, suggesting hybrid states that complicate simple dichotomy (Moore et al., 2015; Witherel et al., 2019).

*In vitro* approaches include macrophage cytokine secretion profiling on defined surface chemistries at set time points (Chang et al., 2008; Brodbeck et al., 2002; Huang et al., 2018; Jannasch et al., 2017a; Zhou et al., 2016; Al-Khoury et al., 2019). Polarization ratios are determined by dividing mean M1 by M2 cytokine percentages across biomaterials (Grotenhuis et al., 2013).

## Key event 3: foreign body giant cell formation and chronic inflammation

A hallmark of the foreign body response is macrophage fusion into foreign body giant cells (FBGCs), defined as cells with three or more nuclei (Anderson et al., 2008; Saleh and Bryant, 2017; Witherel et al., 2018). FBGC formation occurs after implantation of diverse biomaterials and is reported in many case studies in rodents and humans (Larsen et al., 2024; Clauzel et al., 2024; Simoni et al., 2020; Doddridge et al., 2024; Álvarez-Ortega et al., 2021; Fatkhudinov et al., 2019). These cells can persist at the implant surface for the device lifetime, and their long-term presence reflects low-grade chronic inflammation (McNally et al., 2008; Álvarez-Ortega et al., 2021).

Two main hypotheses explain macrophage fusion. First, the lack of FBGCs undergoing apoptosis suggests fusion is an escape mechanism (Brodbeck et al., 2001; Christenson et al., 2005). Thereby, FBGCs may act as a cellular shield integrating into the fibrotic capsule (Trout and Holian, 2020). Second, frustrated phagocytosis of large non-internalizable objects can trigger fusion (Zhao et al., 1991; Kyriakides et al., 2004). However, whether FBGC phagocytic activity exceeds that of mononuclear macrophages remains unclear (Milde et al., 2015).

Sustained FBGC presence at the biomaterial interface correlates with progressive capsule formation. After silicone pad implantation in rats, capsule thickness correlated with FBGC accumulation after four and 12 weeks (Majd et al., 2015; Işıktekin et al., 2025). Similar correlations occurred two and 4 weeks after subcutaneous implantation of nanopatterned metallic glasses in C57BL/6 mice (Shayan et al., 2018). In C57BL/6 mice with drug-releasing spheres, capsule thickness at 4 weeks correlated with FBGC accumulation at 1 week (Morris et al., 2017).

Several *in vitro* models analyze FBGC formation. A standard metric is the fusion index, the number of nuclei within multinucleated giant cells divided by total nuclei per field, applied to PBMC-derived FBGCs across surface chemistries (Chang et al., 2008; Jones et al., 2007; Brodbeck et al., 2002). Additionally, THP-1–derived FBGCs were assessed by May–Grünwald/Giemsa staining to quantify the area percentage of FBGCs (Al-Khoury et al., 2019; Zhou et al., 2015). Murine FBGCs from RAW 264.7 and primary cells were analyzed by F4/80 staining to determine FBGC percentage per field (Majd et al., 2015; Morris et al., 2017). ELISA-based quantification of FBGCs on biomaterial surfaces is under investigation in our lab.

## Key event 4: fibroblast recruitment and myofibroblast differentiation

Macrophage–fibroblast crosstalk via TGF-β1, PDGF, IL-6, and IL-13 promotes chemotaxis and myofibroblast differentiation (Pakshir and Hinz, 2018; Holt et al., 2010; Witherel et al., 2019; Noskovicova et al., 2021a). Precise signaling mechanisms remain incompletely defined. Myofibroblasts are a pathophysiologically relevant activation state marked by excessive collagen production, contraction, and crosslinking (Noskovicova et al., 2021a). Their accumulation drives ECM contracture and may necessitate repeat surgeries for soft-tissue implants (Baker et al., 1981; Coleman et al., 1993).

Alpha-smooth muscle actin (α-SMA) is standard marker of myofibroblast differentiation (Hinz et al., 2001) and linked to fibrotic capsule formation. After silicone implantation in Sprague–Dawley rats, capsule histology showed increased α-SMA–positive cell densities than controls (Liu et al., 2022). In Sprague–Dawley rats, α-SMA–positive myofibroblast counts at the tissue interface correlated with capsule thickness after subcutaneous implantation of functionalized hernia meshes (Yang et al., 2025). In Wistar rats with functionalized or micropatterned silicone pads, capsule thickness correlated with α-SMA–positive myofibroblast abundance (Majd et al., 2015). In C57BL/6 mice implanted with silicones of varying stiffness, capsule thickness correlated with α-SMA–positive myofibroblast density (Noskovicova et al., 2021b).

Feedback in fibroblast–macrophage crosstalk remains a data gap. Fibroblast-secreted mediators enhance FBGC formation, and direct contact enables macrophage fusion on non-permissive substrates (Stewart et al., 2024). Additionally, biomaterial integrin interaction can activate myofibroblast differentiation, independent of paracrine signals (Noskovicova et al., 2021b).

*In vitro* assays include immunofluorescence of fibronectin ED-A and α-SMA in primary human dermal fibroblasts to assess myofibroblast differentiation (Majd et al., 2015; Zhou et al., 2016). A fibroblast outgrowth assay measures outgrowth distance of primary human dermal fibroblasts in coculture with THP-1–derived macrophages on different surface chemistries (Zhou et al., 2016). A fibroblast migration assay quantifies velocity, directness, and forward migration index from live-cell imaging in macrophage–substrate–conditioned medium (Jannasch et al., 2017b).

## Key event 5: extracellular matrix deposition

Fibrosis, including capsule formation, occurs when myofibroblast-mediated collagen synthesis exceeds degradation, causing collagen accumulation (Wynn, 2008). Capsule collagen abundance correlates with thickness. Histochemical collagen density correlated with capsule thickness after micropatterned silicone implantation in Wistar rats (Majd et al., 2015). Similarly, collagen density correlated with thickness after implanting silicones of varying stiffness, in C57BL/6 mice (Noskovicova et al., 2021b).

Intervention studies in Sprague–Dawley rats support causality of collagen synthesis in capsule formation. Subcutaneous implantation of COL1A1 siRNA scaffolds downregulated collagen I (COL1A1) and significantly reduced capsule thickness at 2 and 4 weeks versus plain nanofibers (Rujitanaroj et al., 2013). Silicone implants coated with phosphorylcholine or collagen I synthesis inhibitor halofuginone lowered collagen I and III levels and reduced capsule thickness after 3 months versus uncoated controls (Zeplin et al., 2010a; Zeplin et al., 2010b). Silicone coated with TGF-β inhibitor Tranilast similarly decreased collagen density and capsule thickness from 1 to 12 weeks (Park et al., 2015).

*In vitro* models enable ECM deposition analysis. A co-culture wound model of primary fibroblasts and macrophages embedded in matrix proteins (collagen, fibrin) and exposed to biomaterials enables quantifying collagen I (Western blot) and collagen I/III (histochemical staining) (Jannasch et al., 2019). Sircol and Fastin assays quantify collagen and elastin in rat dermal fibroblast–alveolar macrophage co-cultures in agarose gels contacting polyamide with modified chemistries (Damanik et al., 2014).

## Adverse outcome: fibrotic capsule formation

After preceding key events, a collagenous, largely avascular capsule envelops the biomaterial within 2–4 weeks after implantation (Kyriakides et al., 2004; Anderson et al., 2008). At this stage, the local response stabilizes, and inflammation subsides. However, this fibrotic capsule can isolate the device from surrounding tissue, impair performance, and ultimately render the implant nonfunctional (Sudarsanam et al., 2024).

Although capsule thickness is the benchmark in cited literature, the threshold of capsule formation with observed adverse effect on medical device or patient is decisive for regulation. While the causality is shown, quantitative correlation of capsule formation to clinical adverse outcomes goes beyond this work’s scope.

## Discussion

This perspective initially applies the AOP framework to the FBR. The macrophage-centered AOP maps material–host interactions into key events culminating in fibrotic encapsulation. This structure may guide early screening of biomaterials for low-FBR potential, promote methodology development anchored to key events, and provide assay templates to improve standardization across studies. This aligns with efforts to build AOPs for materials and nanomaterials (Halappanavar et al., 2020; Beasley et al., 2022).

Uncertainties remain and require targeted research. Key event relationships remain unresolved, particularly the transition from macrophage activation to FBGC formation and macrophage–fibroblast crosstalk mechanisms (Witherel et al., 2019). Lymphocyte impact is not addressed due to conflicting data: some studies report macrophage modulation and B-cell chemotaxis (Chang et al., 2009; Doloff et al., 2017), while others indicate minimal involvement (Kyriakides et al., 2022; Rodriguez et al., 2009).

Several studies implicate cytokines, growth factors, and chemokines in capsule formation, yet their spatial and temporal distribution remain incompletely defined (Higgins et al., 2009). Progress from transcriptomics (Liang et al., 2024; Liu et al., 2022; Larsen et al., 2025), proteomics (Schoberleitner et al., 2023), and cell painting (Klinge et al., 2020) is encouraging, but more studies are needed for robust data. Large-scale screenings of biomaterial libraries to uncover quantitative structure–function relationships remain lacking, although initial profiling of polymer libraries is promising (Rostam et al., 2020).

Factors beyond host–material interactions influence FBR and are outside this AOP. Examples include patient-specific variables such as age and radiation, which affect capsular contracture in breast implants (Bachour et al., 2018). Biofilm impact in clinical context is highly controversial (Poppler et al., 2015). Implantation method and site affect FBR. Subcutaneous or peritoneal sites often achieve better outcomes than fat or muscle compartments (Kalashnikov et al., 2025).

Despite uncertainties, novel biomaterials are entering clinical development. To implement them in medical devices, biocompatibility testing must be sensitive to immunomodulation and FBR. As Anderson emphasized, ISO 10993 is a living document, and biocompatibility assessment is evolving with biomaterial features (Anderson, 2016). Therefore, advanced medical devices require appropriate FBR screening of immunomodulating biomaterials to ensure biological safety.

Investigators in biomaterials toxicology are encouraged to address data gaps identified here and build evidence needed for OECD-level acceptance of an AOP for FBR. Integrating spatial omics and imaging is crucial to fully understand and map cellular signals around implants. Coupling *in vitro* and *in vivo* data with clinical outcomes of biomaterials forms an integrated approach and can establish quantitative key event relationships. Curated datasets will accelerate AOP refinement and comparability across studies.

Reaching these goals will enable a defined approach for FBR assessment in regulatory toxicology anchored to an AOP. Thereby, we may predict how material properties influence encapsulation and engineer devices toward a favorable, low-FBR outcome. This will render biocompatibility assessment more mechanistic and human-relevant, supporting safe translation of next-generation biomaterials.

## Data Availability

The original contributions presented in the study are included in the article/Supplementary Material, further inquiries can be directed to the corresponding author.
